# Incomplete ART adherence is associated with higher inflammation in individuals who achieved virologic suppression in the START study

**DOI:** 10.1002/jia2.25297

**Published:** 2019-06-27

**Authors:** Jose R Castillo‐Mancilla, Andrew N Phillips, James D Neaton, Jacqueline Neuhaus, Shweta Sharma, Jason V Baker, Simon Collins, Sharon Mannheimer, Sarah Pett, Veronique Touzeau‐Römer, Mark N Polizzotto, Jens D Lundgren, Edward M Gardner, the INSIGHT START Study Group

**Affiliations:** ^1^ Medicine/Infectious Diseases University of Colorado‐AMC Aurora CO USA; ^2^ Institute for Global Health University College London London United Kingdom; ^3^ School of Public Health University of Minnesota Minneapolis MN USA; ^4^ Hennepin Healthcare Research Institute Minneapolis MN USA; ^5^ School of Medicine University of Minnesota Minneapolis MN USA; ^6^ HIV i‐Base London United Kingdom; ^7^ Harlem Hospital Center Columbia University Medical Center New York NY USA; ^8^ Institute of Clinical Trials and Methodology University College London London United Kingdom; ^9^ Kirby Institute University of New South Wales Sydney Australia; ^10^ AKH Division of Immunology, Allergy and Infectious Diseases University of Vienna Medical School Vienna Austria; ^11^ CHIP Department of Infectious Diseases Rigshospitalet Copenhagen Denmark; ^12^ Denver Health Medical Center Denver CO USA

**Keywords:** adherence, inflammation, antiretroviral therapy, inteleukin‐6, serum amyloid A protein, START study

## Abstract

**Introduction:**

Suboptimal ART adherence, despite HIV viral suppression, has been associated with chronic residual inflammation. Whether this association extends to individuals who initiate ART during early HIV infection remains unknown, which was the objective of this study.

**Methods:**

Plasma levels of interleukin‐6 (IL‐6), high‐sensitivity C‐reactive protein, serum amyloid A protein (SAA), IL‐27, soluble intercellular adhesion molecule‐1, soluble vascular adhesion molecule‐1, D‐dimer and the CD4+/CD8+ T‐cell ratio, were analysed at baseline and eight months after ART initiation in treatment‐naïve participants with HIV and CD4+ T‐cells >500 cells/mm^3^ enrolled in the immediate arm of START. Adherence was assessed by seven‐day self‐report. Multivariable linear regression was utilized to analyse the association between ART adherence and each biomarker at the eight‐month visit in participants who achieved virologic suppression (<50 copies/mL).

**Results:**

We evaluated 1627 participants (422 female) who achieved virologic suppression at the eight‐month visit in the period between 2009 and 2013. Median (IQR) CD4+ T‐cell count before ART was 651 (585, 769) cells/mm^3^. Incomplete adherence was reported in 109 (7%) participants at the eight month visit. After adjusting for covariates, plasma IL‐6 was 1.12 (95% CI, 1.00 to 1.26; p = 0.047) fold higher in participants reporting incomplete versus 100% adherence. A similar association for SAA was observed in an exploratory analysis (1.29 (95% CI 1.04 to 1.60); p = 0.02). No significant differences in other biomarkers were observed.

**Conclusions:**

Incomplete ART adherence was associated with higher IL‐6 levels in individuals who achieved virologic suppression early after ART initiation in START. A potential similar association for SAA requires confirmation. These findings suggest a role for identifying strategies to maximize ART adherence even during virologic suppression. ClinicalTrials.gov number: NCT00867048.

## 1 Introduction

People living with HIV (PLHIV) continue to benefit from antiretroviral therapy (ART) by preventing progression to AIDS 1, 2 and transmission to their partners 3. However, even in the setting of durable and sustained virologic suppression, PLHIV exhibit a phenotype of enhanced residual inflammation, immune activation and coagulopathy 4 that is predictive of serious non‐AIDS events, including cardiovascular disease, non‐AIDS‐related malignancies and all‐cause mortality 5, 6, 7. A wide variety of potential mechanisms driving this phenomenon have been proposed, including a high prevalence of specific lifestyle behaviours such as smoking, illicit substance use and obesity (all of which are common among PLHIV) 8, 9, and other factors such as microbial translocation 6, viral co‐infections (i.e. cytomegalovirus, hepatitis C virus) 10, 11 and HIV persistence in lymphoid tissues 12. To date, efforts to reverse residual inflammation and immune activation in treated HIV infection (e.g. ART intensification, anti‐inflammatories, treating co‐infections) have proven moderately successful 13, 14, 15, 16, 17. Thus, a better understanding of the pathogenesis of residual inflammation in HIV is needed in order to develop effective interventions to reduce it and, potentially, improve clinical outcomes.

Recent studies have demonstrated that suboptimal (i.e. less than 100%) ART adherence could be a significant contributing factor to the chronic residual inflammation, coagulopathy and immune activation observed in PLHIV even if it is sufficient to achieve and sustain plasma viral suppression through routinely available assays 18, 19, 20. These associations have been identified in PLHIV who are on chronic ART 18, 20 and who have recently initiated ART 19. However, these studies included participants who mostly initiated ART with advanced disease (i.e. CD4^+^ T‐cells <200 or <350 cells/mm^3^), based on the treatment guidelines that were prevalent at the time. Whether variations in ART adherence, beyond virologic suppression, are also associated with heightened chronic residual inflammation, immune activation and coagulopathy in early treated HIV infection remains unknown.

To address the above‐mentioned gap, we evaluated the association between incomplete ART adherence with residual inflammation, immune activation, coagulopathy and vascular inflammation in treatment naïve PLHIV who initiated ART with CD4^+^ T cells >500 cells/mm^3^ and achieved viral suppression in the immediate arm of the Strategic Timing of Antiretroviral Treatment (START) study. Based on our previous findings in the SMART study 20, we hypothesized that plasma biomarkers of inflammation and coagulopathy (interleukin (IL)‐6 and D‐dimer) would be higher in participants who achieved virologic suppression but who reported incomplete versus 100% adherence. In addition, we aimed to explore the association between ART adherence and additional biomarkers of inflammation, immune activation and vascular inflammation that were assayed in the study population.

## 2 Methods

### 2.1 Participants

Within the period between April 2009 through December 2013, the START study enrolled PLHIV who were 18 years of age or older and who were ART naïve, had no history of an AIDS‐defining illness, and had CD4^+^ T cells >500 cells/mm^3^, as previously reported 21. To be included in this analysis, participants were required to: (1) have been randomized to the immediate treatment arm of START; (2) have achieved viral suppression (<50 copies/mL) while on ART at the eight‐month visit, and; (3) have concomitant adherence data and plasma biomarkers available at the eight‐month visit. Each participating site obtained study approval by their corresponding local institutional review board or ethics committee, and written informed consent was obtained from all participants prior to any study procedures.

### 2.2 Adherence assessment

ART adherence was measured by self‐report at one, four and eight months after ART initiation using a case report form modelled after the Terry Beirn Community Programs for Clinical Research on AIDS (CPCRA) Antiretroviral Medication Self‐Report Form 065‐BAS‐2, which uses a seven‐day global recall, where participants respond whether they took “all my pills every day,” “most of my pills,” “about one‐half of my pills,” “very few of my pills,” or “none of my pills” for each specific pill in their ART regimen. This adherence measure has been previously validated to predict viral suppression and the development of viral rebound 22, 23, and has been found to be associated with heightened inflammation in participants with viral suppression in the SMART study 20. ART adherence at the eight‐month visit was labelled as “incomplete” if a participant reported any option other than taking “all of my pills everyday” for any antiretroviral medication, and as “100%” if a participant reported taking “all of my pills everyday” for all ART medications, as previously described 20. A supplementary sensitivity analysis, in which a participant was labelled to have incomplete adherence if s/he reported <100% adherence to any ART medication at any of the month one, four or eight visits, was also performed to assess the robustness of our findings.

### 2.3 Biomarkers of systemic inflammation, vascular inflammation and coagulopathy

Plasma samples were collected at baseline and at the eight‐month visit, and were stored at −70°C in a central laboratory. In these samples, the following seven biomarkers reflecting (1) systemic inflammation; (2) immune activation; (3) vascular inflammation, and; (4) coagulopathy, were quantified by blinded researchers: (1) IL‐6, high‐sensitivity C‐reactive protein (hsCRP) and serum amyloid A protein (SAA); (2) IL‐27; (3) soluble intercellular adhesion molecule‐1 (sICAM) and soluble vascular adhesion molecule‐1 (sVCAM), and; (4) D‐dimer. IL‐6 was quantified using a high‐sensitivity enzyme‐linked immunosorbent assay (R&D Systems); IL‐27 was quantified using immunoassay (MesoScale, MSD); D‐dimer was quantified using the VIDAS system (BioMerieux); hsCRP, SAA, sICAM and sVCAM were quantified using the Vascular Injury II Panel (MesoScale, MSD), as previously described 24. CD4^+^ and CD8^+^ T‐cell subsets were determined by flow cytometry at each site's clinical laboratory.

### 2.4 Statistical analysis

Baseline participant demographic characteristics were summarized through the appropriate statistical measures. Adherence was dichotomized as incomplete (<100%) or 100%. For two of the biomarkers, IL‐6 and D‐dimer, there was a pre‐specified hypothesis that they would be associated with sub‐optimal adherence based on our previous work 20. For the other biomarkers, these were considered exploratory analyses. The plasma concentrations of the biomarkers of inflammation, immune activation, vascular inflammation and coagulopathy (except the CD4^+^/CD8^+^ T‐cell ratio) were natural log (ln)‐transformed in order to address data skewing. This was followed by univariable and multivariable linear regression analysis aimed at assessing the association between ART adherence and the log‐transformed biomarker concentrations and the CD4^+^/CD8^+^ T‐cell ratio at the eight‐month visit in participants who achieved virologic suppression to <50 copies/mL. These analyses were adjusted for the following baseline characteristics: sex, age, race, biomarker concentrations, level of education, HIV risk factor, region of enrolment, hepatitis B (defined as a positive hepatitis B surface antigen) or hepatitis C (defined as a positive hepatitis C antibody) co‐infection, body mass index (BMI), and smoking. These variables have previously been associated with inflammation and coagulopathy in other studies 25, 26, including START 24, and/or with suboptimal adherence (e.g. African American race 27, smoking 28, treatment in resource‐limited settings 29). Data are presented as fold differences in biomarker concentrations and in the CD4^+^/CD8^+^ T‐cell ratio in individuals who reported incomplete versus 100% adherence at the eight‐month visit. All statistical analyses were performed using SAS version 9.4. For the biomarkers included in the exploratory analyses we used the non‐parametric rank sum method proposed by O'Brien 30, 31, which provides a single overall test of the null hypothesis of no difference between the two adherence categories for these five biomarkers (excluded CD4^+^/CD8^+^ ratio). *p*‐values were not adjusted for multiple comparisons. A value of *p* < 0.05 was considered to be statistically significant.

## 3 Results

### 3.1 Study participants

From a total of 4684 participants enrolled from 215 sites in 35 countries in the START study, 2325 were randomized to immediate ART. Of these, 1627 (70%) were virologically suppressed to <50 copies/mL and had available adherence data and biomarkers at the eight‐month visit (Figure S1). Baseline demographics included median age of 36 (interquartile range (IQR) 29, 44) years, median CD4^+^ T‐cell count of 651 (IQR 585, 769) cells/mm^3^ and a median HIV viral load of 13,123 (IQR 3331, 42,169) copies/mL before initiation of ART. Additional demographic characteristics of these participants upon enrolment, according to adherence status, are presented in Table 1.

**Table 1:** Demographic characteristics of the immediate ART arm participants in START with HIV RNA <50 copies/mL at the eight‐month visit, categorized by ART adherence at month 8 (n=1627)

Characteristic	Incomplete adherence n=109	100% Adherence n=1518
	n (%) or median (IQR)	
Female sex	24 (22)	398 (26)
Age (years)	32 (27, 40)	36 (29, 44)
Time since diagnosis (years)	1.1 (0.4, 3.0)	0.9 (0.3, 2.8)
Race		
Black	31 (28)	448 (30)
Hispanic	24 (22)	191 (13)
Asian	10 (9)	139 (9)
White	35 (32)	683 (45)
Other	9 (8)	57 (4)
ART class at eight‐month visit^a^		
NNRTI‐based	77 (71)	1086 (72)
Efavirenz	74 (68)	1010 (67)
Rilpivirine	3 (3)	71 (5)
Nevirapine/etravirine	0 (0)	5 (<1)
b/PI‐based	22 (20)	337 (22)
Atazanavir	14 (13)	181 (12)
Darunavir	7 (6)	126 (8)
Lopinavir	1 (<1)	19 (1)
Fosamprenavir	0 (0)	11 (<1)
INSTI‐based^b^	9 (8)	76 (5)
Other/multiclass	1 (<1)	19 (1)
Education level		
Less than high school (less than year 12 or “A” level)	33 (30)	448 (30)
High school or equivalent (year 12 or “A” level)	22 (20)	334 (22)
Completed vocational training	11 (10)	145 (10)
Some college/some university	18 (17)	262 (17)
Bachelor's/university/TAFE degree	20 (18)	248 (16)
Any post‐graduate education	5 (5)	81 (5)
HIV exposure		
IDU	1 (1)	22 (1)
MSM	73 (67)	844 (56)
Heterosexual	33 (30)	572 (38)
Other	2 (2)	80 (5)
Region		
Africa	16 (15)	341 (22)
Latin America	37 (34)	382 (25)
Europe/Israel	26 (24)	511 (34)
United States	19 (17)	123 (8)
Australia	2 (2)	36 (2)
Asia	9 (8)	125 (8)
HBV infection	0 (0)	44 (3)
HCV infection	4 (4)	51 (3)
BMI at baseline (kg/m^2^)	24.4 (22.3, 29.3)	24.5 (22.0, 27.8)
Current smoker (baseline)	43 (39)	476 (32)
CD4^+^ T cells at baseline (cells/mm^3^)	658 (596, 760)	649 (585, 769)
HIV RNA at baseline (copies/mL)	9803 (3017, 34,178)	13,411 (3382, 42,717)

ART, antiretroviral therapy; BMI, body mass index; HBV, hepatitis B virus; HCV, hepatitis C virus; IDU, injection drug users; INSTI, integrase strand‐transfer inhibitor; IQR, inter‐quartile range; MSM, men who have sex with men; NNRTI, non‐nucleoside reverse transcriptase inhibitor; PI, protease inhibitor; VL, viral load.

^a^All participants received a dual nucleoside/nucleotide reverse transcriptase inhibitor backbone; ^b^INSTI‐based therapy was raltegravir based; only one participant was prescribed elvitegravir in the 100% adherence group.

### 3.2 ART adherence

Among the 1627 participants included in the analysis, 109 (7%) reported incomplete adherence at the eight‐month visit (Table 1). Overall, the proportions of participants with incomplete adherence were similar according to ART class (Table 1). Most (84%) participants in the incomplete adherence group reported taking “most of my pills” for at least one of their ART drugs, while 11 (10%) participants in this group reported taking “none of my pills” for all their prescribed ART, 9 of whom were on an efavirenz‐based regimen. When the definition of incomplete adherence was expanded to include any report of <100% adherence at any of the one, four or eight months visits, the number of participants who had incomplete adherence increased to 276 (17%) of the study population.

### 3.3 Biomarkers of systemic inflammation, vascular inflammation and coagulopathy

The plasma concentrations of biomarkers of inflammation, immune activation, coagulopathy and vascular inflammation at baseline and at the eight‐month visit are presented in Table 2. Overall, all the measured plasma biomarkers, except IL‐27, decreased between the baseline and the eight‐month visits, showing statistically significant differences. The CD4^+^/CD8^+^ T‐cell ratio increased from 0.66 (IQR 0.49, 0.89) at baseline to 0.98 (IQR 0.74, 1.28) at the eight‐month visit (*p* < 0.0001). Baseline concentrations for all biomarkers were significantly associated with the eight‐month concentrations in an adjusted analysis (range of correlation coefficients 0.40 to 0.78, *p* < 0.0001; data not shown). There were no statistically significant associations between baseline biomarker levels and adherence at eight months (data not shown).

**Table 2:** Biomarkers of inflammation, immune activation, vascular inflammation and CD4^+^/CD8^+^ T‐cell ratio before and eight‐months after ART initiation (sample restricted to those with values at both time points in the immediate arm of START)

Biomarker		Baseline	Eight‐month visit	
	n^a^	Median (IQR)	Median (IQR)	*p*‐value
IL‐6 (pg/mL)	1626	1.39 (0.98, 2.15)	1.24 (0.84, 1.94)	<0.0001
hsCRP (μg/mL)	1626	1.86 (0.76, 4.15)	1.82 (0.74, 4.66)	0.005
SAA (mg/L)	1626	4.8 (2.6, 9.1)	3.8 (2.1, 7.8)	<0.0001
IL‐27 (pg/mL)	1626	244 (123, 506)	240 (111, 534)	0.1
sICAM (ng/mL)	1626	543 (424, 693)	477 (369, 611)	<0.0001
sVCAM (ng/mL)	1626	719 (556, 928)	555 (439, 884)	<0.0001
D‐dimer (μg/mL)	1616	0.32 (0.22, 0.49)	0.27 (0.19, 0.43)	<0.0001
CD4^+^/CD8^+^ ratio	1606	0.66 (0.49, 0.89)	0.98 (0.74, 1.28)	<0.0001

*p*‐values are from Wilcoxon Signed rank test, based on individuals who had available biomarker data at both baseline and eight month visits. ART, antiretroviral therapy; hsCRP, high‐sensitivity C‐reactive protein; IL‐27, interleukin 27; IL‐6, interleukin 6; IQR, interquartile range; SAA, serum amyloid A protein; sICAM, soluble intercellular adhesion molecule‐1; sVCAM, soluble vascular adhesion molecule‐1.

a Sample sizes may be smaller than what is reported in Table 1 due to missing data at the eight‐month visit.

### 3.4 Association between ART adherence and biomarkers

The plasma concentrations of biomarkers of inflammation, immune activation, vascular inflammation, coagulopathy and the CD4^+^/CD8^+^ T‐cell ratio, according to ART adherence category in the participants who were virologically suppressed at the eight‐month visit, are shown in Table 3. Overall, the plasma concentrations of biomarkers were higher among the participants who reported incomplete versus 100% adherence, except for sVCAM and D‐dimer (Table 3). In the univariable analysis, plasma concentrations of IL‐6 were 1.14 (95% CI, 1.00 to 1.31; *p* = 0.04) fold higher, and of D‐dimer were 0.90 (95% CI, 0.79 to 1.02; *p* = 0.10) fold lower, in participants who reported incomplete versus 100% adherence respectively (Table 3). These remained statistically significant for IL‐6 after adjusting for sex, age, race, baseline biomarker concentrations, level of education, HIV risk factor, region of enrolment, viral hepatitis co‐infection, BMI and smoking, with concentrations that were 1.12 (95% CI, 1.00 to 1.26; *p* = 0.047) fold higher in participants who reported incomplete versus 100% adherence (Table 3). Considering the biomarkers in the exploratory analysis, the *p*‐value for the O'Brien global test was *p* = 0.35. In this analysis, plasma concentrations of SAA were 1.25 (95% CI, 0.99 to 1.57; *p* = 0.06) fold higher in participants who reported incomplete versus 100% adherence in the univariate analysis, and became statistically significant after adjusting for covariates, with 1.29 (95% CI, 1.04 to 1.60; *p* = 0.02) fold higher plasma concentrations among participants who reported incomplete versus 100% adherence respectively (Table 3). No significant differences were observed in the remaining biomarkers or in the CD4^+^/CD8^+^ T‐cell ratio between participants who reported incomplete versus 100% adherence at the eight‐month visit (Table 3).

**Table 3:** Distribution of biomarker concentrations and CD4^+^/CD8^+^ T‐cell ratio at the eight‐month visit according to ART adherence category, with fold difference between ART adherence categories in PLHIV who achieved HIV viral load <50 copies/mL at the eight‐month visit in the immediate arm of START

Biomarker	Incomplete adherence		100% Adherence^a^		Unadjusted			Adjusted^b^		
	n	Median (IQR)	n	Median (IQR)	Fold difference	95% CI	*p*‐value	Fold difference	95% CI	*p*‐value
Biomarkers for which there was a pre‐specified hypothesis based on previous data										
IL‐6 (pg/mL)	109	1.34 (0.95, 2.10)	1518	1.23 (0.84, 1.92)	1.14	1.00 to 1.31	*0.04*	1.12	1.00 to 1.26	*0.047*
D‐dimer (μg/mL)	109	0.25 (0.18, 0.38)	1513	0.28 (0.19, 0.43)	0.90	0.79 to 1.02	0.10	0.96	0.87 to 1.07	0.47
Exploratory analyses of other biomarkers^c^										
hsCRP (μg/mL)	109	2.32 (0.75, 5.65)	1518	1.81 (0.74, 4.58)	1.16	0.88 to 1.51	0.28	1.20	0.96 to 1.50	0.11
SAA (mg/L)	109	4.59 (2.72, 9.71)	1518	3.72 (2.07, 7.73)	1.25	0.99 to 1.57	0.06	1.29	1.04 to 1.60	*0.02*
IL‐27 (pg/mL)	109	265 (109, 546)	1518	239 (111, 533)	0.98	0.74 to 1.28	0.85	1.00	0.84 to 1.19	0.98
sICAM (ng/mL)	109	487 (366, 625)	1518	476 (370, 610)	1.02	0.93 to 1.11	0.72	1.01	0.94 to 1.08	0.86
sVCAM (ng/mL)	109	535 (452, 690)	1518	556 (437, 690)	1.00	0.92 to 1.08	0.92	1.00	0.93 to 1.07	0.97
CD4^+^/CD8^+^ ratio	109	0.94 (0.71, 1.25)	1512	0.98 (0.74, 1.29)	0.96	0.87 to 1.05	0.32	0.96	0.90 to 1.02	0.22

hsCRP, high‐sensitivity C‐reactive protein; IL‐27, interleukin 27; IL‐6, interleukin 6; IQR, interquartile range; PLHIV, people living with HIV; SAA, serum amyloid A protein; sICAM, soluble intercellular adhesion molecule‐1; sVCAM, soluble vascular adhesion molecule‐1.

^a^100% adherence defined as no report of any missed doses for any drug in the preceding seven‐day period; ^b^models were adjusted for covariates including sex, age, race, baseline biomarker concentrations (or CD4^+^/CD8^+^ ratio), level of education, HIV risk factor, region of enrolment, viral hepatitis co‐infection, body mass index and smoking; ^c^O'Brien test overall *p* = 0.35.

In the sensitivity analysis, when the definition of incomplete ART adherence was expanded to include any report of <100% adherence at any of the one, four or eight months visits, the participants categorized to have incomplete adherence had 1.10 (1.01 to 1.19; *p* = 0.02) fold higher plasma concentrations of IL‐6, 1.01 (0.94 to 1.08; *p* = 0.85) fold higher plasma concentrations of d‐dimer, and 1.15 (0.99 to 1.33; *p* = 0.06) fold higher plasma concentrations of SAA at the eight‐month visit, respectively, compared to participants who reported 100% adherence (Table 4, adjusted analysis). No significant differences in the other biomarkers or the CD4^+^/CD8^+^ T‐cell ratio were observed using this broader definition of incomplete adherence (Table 4). When participants who reported taking “none of my pills” (n=11) were removed from the analysis, those with imperfect adherence had 1.11 (0.98 to 1.25; *p* = 0.10) fold higher plasma concentrations of IL‐6 (in an adjusted analysis) and 0.86 (0.75 to 0.99; *p* = 0.03) fold lower plasma concentrations of d‐dimer when compared with participants who reported 100% adherence (unadjusted analysis, results of the adjusted analysis were not significant – data not shown).

**Table 4:** Sensitivity analysis using an expanded definition of incomplete ART adherence^a^ presenting the distribution of biomarker concentrations and CD4^+^/CD8^+^ T‐cell ratio at the eight‐month visit according to ART adherence category, with fold difference between ART adherence categories in PLHIV who achieved HIV viral load <50 copies/mL at the eight‐month visit in the immediate arm of START

Biomarker	Incomplete adherence		100% Adherence^b^		Unadjusted			Adjusted^c^		
	n	Median (IQR)	n	Median (IQR)	Fold difference	95% CI	*p*‐value	Fold difference	95% CI	*p*‐value
Biomarkers in the pre‐specified hypothesis based on previous data										
IL‐6 (pg/mL)	276	1.37 (0.92, 2.29)	1351	1.22 (0.83, 1.87)	1.16	1.07 to 1.26	*0.001*	1.10	1.01 to 1.19	*0.02*
D‐dimer (μg/mL)	276	0.26 (0.18, 0.42)	1347	0.28 (0.19, 0.43)	0.97	0.89 to 1.06	0.44	1.01	0.94 to 1.08	0.85
Exploratory analyses of other biomarkers										
hsCRP (μg/mL)	276	2.05 (0.83, 5.37)	1351	1.81 (0.72, 4.52)	1.11	0.93 to 1.32	0.26	1.09	0.94 to 1.27	0.25
SAA (mg/L)	276	4.43 (2.27, 9.35)	1351	3.67 (2.05, 7.63)	1.12	0.96 to 1.31	0.15	1.15	0.99 to 1.33	0.06
IL‐27 (pg/mL)	276	242 (117, 484)	1351	239 (109, 539)	0.94	0.78 to 1.12	0.48	1.02	0.91 to 1.14	0.77
sICAM (ng/mL)	276	482 (371, 609)	1351	475 (367, 611)	1.02	0.96 to 1.08	0.50	1.01	0.96 to 1.06	0.64
sVCAM (ng/mL)	276	544 (436, 679)	1351	558 (440, 692)	1.00	0.94 to 1.05	0.91	1.01	0.96 to 1.06	0.71
CD4^+^/CD8^+^ ratio	275	1.02 (0.72, 1.33)	1346	0.97 (0.74, 1.27)	1.02	0.96 to 1.09	0.43	1.01	0.96 to 1.05	0.79

hsCRP, high‐sensitivity C‐reactive protein; IL‐27, interleukin 27; IL‐6, interleukin 6; IQR, interquartile range; PLHIV, people living with HIV; SAA, serum amyloid A protein; sICAM, soluble intercellular adhesion molecule‐1; sVCAM, soluble vascular adhesion molecule‐1.

^a^Incomplete adherence defined as any report of <100% adherence at any of the month one, four or eight visits; ^b^100% adherence defined as no report of any missed doses for any drug in the preceding seven‐day period; ^c^models were adjusted for covariates including sex, age, race, baseline biomarker concentrations (or CD4^+^/CD8^+^ ratio), level of education, HIV risk factor, region of enrolment, viral hepatitis co‐infection, body mass index and smoking.

## 4 Discussion

This study identified an inverse association between ART adherence and plasma concentrations of IL‐6 in participants who achieved virologic suppression after eight months of ART in the immediate arm of the START study. However, it did not confirm this association for D‐dimer as originally hypothesized. In addition, this analysis describes a potential novel similar association with SAA in the exploratory analyses. These results for IL‐6, while moderate in size (i.e. 12% difference), are consistent with the range of concentrations from previous studies (including SMART and START) where IL‐6 was found to be predictive of mortality and adverse events in HIV 24, 26, 32, 33. They are also consistent with prior findings 18, 19, 20 evaluating the association of suboptimal adherence and inflammation, and expand it to individuals who initiated ART with high CD4^+^ T‐cell counts (>500 cells/mm^3^). This is of particular importance given the significant associations of heightened systemic inflammation and coagulopathy with increased risk for AIDS events, SNAEs and death observed in the START study 21. Collectively, our findings re‐emphasize the biologic importance that optimal adherence could have on maximizing the therapeutic benefit of ART, even in individuals who start ART with high CD4^+^ T cells, in whom advanced immunodeficiency has not been established 34.

As noted, our results confirm previous observations in diverse populations where incomplete adherence had a similar association with inflammation and immune activation. For example, data from the MACS cohort demonstrated that <100% (and in particular <85% adherence) self‐reported adherence to ART (using four‐day and six‐month recall) was associated with 11% to 21% higher plasma concentrations of IL‐10, tumour necrosis factor‐alpha, IL‐6, IL‐2, interferon‐gamma and CRP in 2816 person‐visits from 912 virologically suppressed (<50 copies/mL) men living with HIV 18. Similarly, an analysis of 270 treatment‐naïve individuals who achieved viral suppression (<400 copies/mL) after six months of ART in Uganda found that a 10% increase in average adherence, measured using the Medication Event Monitoring System (MEMS), was associated with a 3% to 15% decrease in plasma levels of soluble CD14 (*p* = 0.03), D‐dimer (*p* = 0.02) and IL‐6 (*p* < 0.0001) 19. Lastly, similar findings were identified in the SMART study, where <100% self‐reported ART adherence (using seven‐day recall) was associated with 9% and 11% higher plasma concentrations of IL‐6 (*p* = 0.02) and D‐dimer (*p* = 0.03) respectively in over 2700 HIV‐infected participants who were virologically suppressed (<200 copies/mL) upon enrolment 20. Of note, all of these associations (and those in our current study), remained significant after adjusting for available demographic and clinical covariates that have previously been associated with biomarkers of inflammation and coagulopathy. In addition, these studies were conducted in diverse observational and research cohorts vastly representative of real‐world clinical populations. This is in comparison to recent studies in participants who had very long‐standing and sustained viral suppression, where cumulative drug exposure was not associated with biomarkers of inflammation 35.

While treated HIV infection has been associated with persistent elevations of IL‐6 and CRP in multiple cohorts and clinical trials 5, 26, 36, the role of SAA in this setting remains poorly understood. Although the association between SAA and suboptimal adherence was identified in the exploratory analysis of additional biomarkers, a *p* = 0.35 in the O'Brien test suggests that the null hypothesis of no differences in biomarkers by adherence category could not be rejected, thus there should be caution in interpreting these results. However, these findings are biologically consistent with our IL‐6 results and suggest that variable ART adherence may be most impactful through a unified inflammatory pathway that involves these biomarkers (and could possibly also include CRP), in comparison to other networks of inflammation or defective adaptive immunity (i.e. CD4^+^/CD8^+^T‐cell ratio) that are associated with adverse outcomes in HIV infection 34, 37. SAA has been traditionally regarded as an acute phase reactant lipoprotein 38, which is mainly synthesized by the liver and has been found to correlate with CRP in the setting of acute 39 and chronic inflammation 40, and to predict cardiovascular outcomes 41. Recently, it has been proposed that SAA could be a mechanistic link between obesity and cardiovascular disease, by virtue of promoting inflammation at the level of the adipose tissue, leading to insulin resistance 42, 43 and dyslipidemia 42, and that it can improve with weight loss 44 and statin therapy 42, 45. In the HIV arena, SAA has been proposed as a marker of very acute HIV infection and to have early antiviral properties 46. Comparatively, SAA has been found to correlate with other biomarkers of inflammation including CRP, IL‐6 and IL‐8 in chronic HIV infection 47, and with the development of opportunistic disease 48, but not mortality or early treatment discontinuation, in the SMART study 26. In START, SAA was also found to be strongly correlated with biomarkers of vascular inflammation including hsCRP, sICAM and sVCAM, which translated into an increased risk of AIDS‐related events in participants who had higher concentrations of SAA 24. Further studies to confirm our findings, and to better understand the clinical associations between ART adherence and SAA in treated HIV infection, are required.

In contrast to our findings in START and other cohorts, recent studies that have evaluated incomplete ART adherence by means of alternative dosing of oral regimens, such as four days on/three days off 49 or weekends off ART 50 in virologically suppressed adults and children, have not demonstrated any impact in biomarkers of inflammation or coagulopathy. In fact, D‐dimer showed a trend towards lower plasma concentrations in children and young adults randomized to weekends off efavirenz‐based ART in one of these studies 50, which was unexpected, but similar to our findings of lower D‐dimer in sub optimally adherent participants in START. Among the possible explanations for these different findings is that the patients selected for inclusion in these studies constituted a population with long‐standing virologic suppression, which differs from the study populations evaluated in previous studies where adherence and inflammation were associated 18, 19, 20. Alternatively, the periods off ART (and their consequential decrease in drug exposure) that were instituted in these studies were predictable and cyclic, in comparison with the more erratic and irregular variations in ART adherence that are expected (and have been observed) in clinical and research cohorts. These differences emphasize the need for further research in this area, including the analysis of biomarkers of inflammation and coagulopathy in randomized studies where participants have been assigned to less‐than‐daily dosing (NCT03256422) 51 or have been simplified to maintenance with two‐drug ART regimens.

Several mechanisms to explain our observed association between incomplete ART adherence and higher inflammation have been proposed. Among them are the possibility of subclinical viral replication below the limit of detection of conventional assays or the occurrence of intermittent episodes of measurable (but missed) viraemia that lead to bursts of inflammation, but which cannot be captured in the time points between clinical or research visits in individuals who are not fully adherent 18. An additional potential explanation could be that patients who are 100% adherent to their ART could also be more adherent to non‐HIV medications with an anti‐inflammatory effect (i.e. statins or aspirin), and that they could also practice overall healthier behaviours that lead to a more pronounced reduction in inflammation (i.e. less likely to smoke, more likely to exercise and eat a healthier diet). While our analysis controlled for smoking and BMI, we did not capture any additional information on these or other potential confounding factors associated with a healthier lifestyle, which may also be associated with lower levels of inflammation. Future studies focused on interventions aimed at reducing chronic residual inflammation in treated HIV infection should include measures of ART adherence and determinants of a healthy lifestyle in order to fully understand these interactions.

Along with the possible explanatory mechanisms, the potential clinical findings derived from our observations merit specific considerations. As ART has become easier to take in the modern era, it has also become more pharmacologically forgiving, allowing for as low as 80% to 85% adherence to achieve and sustain an undetectable viral load 52. Thus, achieving and sustaining viral suppression using clinically available assays (i.e. <50 or <20 copies/mL) is not a surrogate for perfect adherence. Of note, our findings were attenuated when the small subset of participants who reported full non‐adherence in the last seven days were removed, although the directionality of the association remained unchanged. This finding is similar to previous observations in the MACS, where <85% adherence (vs. 85% to 99% adherence) was the main driver of heightened inflammation for most biomarkers. To date, the biological and clinical consequences that develop in the range between “suppressive” and optimal adherence have only been partially evaluated. In particular, whether an improvement in ART adherence, through a proven behavioural intervention or by means of easier dosing (i.e. use of long‐acting injectable ART), can be translated into a reduction in inflammation in the setting of virologic suppression remains unknown, but should be evaluated. In this context, an increase in ART adherence (even if not perfect) that is coupled with a reduction in inflammation would be highly clinically impactful, as it could modify our current treatment focus and encourage patients and providers to strive for the highest possible adherence, especially if all the negative consequences of incomplete adherence are fully recognized.

Our study offers several strengths that should be emphasized. First, it is based on longitudinal data from a large, racially/ethnically diverse sample size enrolled within a multinational clinical trial. Second, the biomarker profile that was analysed is broad and includes markers of vascular inflammation, which had not been previously evaluated in the context of suboptimal ART adherence beyond viral suppression. Lastly, we were able to confirm the association between adherence and inflammation in individuals with suppressed HIV viraemia and early HIV disease and preserved CD4^+^ T‐cell counts. Among the limitations of our analysis are that the size of the association is moderate in magnitude, and that we were unable to assess any potential association of suboptimal ART adherence with clinical endpoints, mostly due to the overall low frequency of events in the START study. In addition, self‐reported adherence has limitations such as recall and social desirability bias 53, which could have lessened the effect size of our findings. Furthermore, higher adherence, in the context of a clinical trial, may also reflect an overall healthier life style 54. Similarly, this analysis could not determine whether the association between ART adherence and inflammation extended beyond the eight‐month time point, or whether it extended to changes in biomarkers of monocyte activation (not evaluated in START). Finally, we could not assess whether our findings were ART‐class specific given the small proportion of participants on an integrase‐based regimen. Future studies focusing on objective measures of ART adherence and their association with clinical endpoints are needed, including the effects of the duration and severity of non‐adherence on chronic inflammation in PLHIV.

## 5 Conclusions

In summary, we identified an association between incomplete ART adherence and systemic inflammation in the setting of viral suppression in PLHIV who initiated treatment with high CD4^+^ T‐cell counts. This confirms previous findings in other cohorts and suggests it may be important to optimize adherence even during virologic suppression as a possible intervention to improve residual inflammation and reduce morbidity and mortality in HIV disease.

## Competing interests

The content is solely the responsibility of the authors and does not necessarily represent the official views of the National Institutes of Health. A.P. received speaker fees for two presentations in 2015. Other authors reported no conflicts of interest.

## Authors’ contributions

JCM formulated the primary hypotheses, led the conception of this analysis and interpretation of the results; presented the results at an international conference; wrote the first manuscript draft and performed all the edits for all the subsequent drafts. ANP co‐led the conception of this analysis, performed the data and statistical analysis and interpretation, generated tables and made substantial edits and critical revisions to the manuscript. JDN, JN participated in the study design, data interpretation and made substantial edits and critical revisions to the manuscript. SS participated in the study design, data interpretation and made substantial edits and critical revisions to the manuscript. JVB led the biomarker analysis, participated in the study design, data interpretation and made substantial edits and critical revisions to the manuscript. SC was part of the community advisory board for the study, participated in the study design, data interpretation and made substantial edits and critical revisions to the manuscript. SM, SP, VTR, MNP, JDL participated in the study design, data interpretation and made substantial edits and critical revisions to the manuscript. EMG co‐led the conception of this analysis and interpretation of the results and made substantial edits and critical revisions to the manuscript.

## Supporting information


**Figure S1.** Flow diagram of participants included in the analysis (word file).
