# Supplementary material for: Incomplete ART adherence is associated with higher inflammation in individuals who achieved virologic suppression in the START study
Source: J Int AIDS Soc. 2019 Jun 27;22(6):e25297. doi: 10.1002/jia2.25297 (PMC6597899; doi:10.1002/jia2.25297)
Supplement: Supplementary file 1 — Figure S1. Flow diagram of participants included in the analysis (word file). [file JIA2-22-e25297-s001.docx]

**Supplemental Figure.** Flow diagram of the participant selection for analysis of the association between ART adherence and biomarkers of systemic inflammation, vascular inflammation and coagulopathy in START.

Included in analysis

(n=1,627)

Excluded (n=698)

HIV VL <50 copies/mL on ART

PLUS

Adherence data + Biomarkers at 8-month visit

Enrolled in START (n=4,684)

Randomized to immediate ART (n=2,325)
